# Genome-Wide Marker Data-Based Comparative Population Analysis of Szeklers From Korond, Transylvania, and From Transylvania Living Non-Szekler Hungarians

**DOI:** 10.3389/fgene.2022.841769

**Published:** 2022-03-28

**Authors:** Valerián Ádám, Zsolt Bánfai, Katalin Sümegi, Gergely Büki, András Szabó, Lili Magyari, Attila Miseta, Miklós Kásler, Béla Melegh

**Affiliations:** ^1^ Department of Medical Genetics, Medical School, University of Pécs, Pécs, Hungary; ^2^ Szentágothai Research Centre, University of Pécs, Pécs, Hungary; ^3^ Department of Laboratory Medicine, Medical School, University of Pécs, Pécs, Hungary; ^4^ National Institute of Oncology, Budapest, Hungary

**Keywords:** population genetics, identical by descent, Transylvania, Szeklers, genome-wide data

## Abstract

Genome-wide genotype data from 48 carefully selected population samples of Transylvania-living Szeklers and non-Szekler Hungarians were analyzed by comparative analysis. Our analyses involved contemporary Hungarians living in Hungary, other Europeans, and Eurasian samples counting 530 individuals altogether. The source of the Szekler samples was the commune of Korond, Transylvania. The analyzed non-Szekler Hungarian samples were collected from villages with a history dating back to the era of the Árpád Dynasty. Population structure by principal component analysis and ancestry analysis also revealed a great within-group similarity of the analyzed Szeklers and non-Szekler Transylvanian Hungarians. These groups also showed similar genetic patterns with each other. Haplotype analyses using identity-by-descent segment discovering tools showed that average pairwise identity-by-descent sharing is similar in the investigated populations, but the Korond Szekler samples had higher average sharing with the Hungarians from Hungary than non-Szekler Transylvanian Hungarians. Average sharing results showed that both groups are isolated compared to other Europeans, and pointed out that the non-Szekler Transylvanian Hungarian inhabitants of the investigated Árpád Age villages are more isolated than investigated Szeklers from Korond. This was confirmed by our autozygosity analysis as well. Identity-by-descent segment analyses and 4-population tests also confirmed that these Hungarian-speaking Transylvanian ethnic groups are strongly related to Hungarians living in Hungary.

## Introduction

Transylvania is a historical region located in today’s Romania, which is bordered on the east and south by the Carpathian Mountains and the Apuseni Mountains on the west. From the 1st century BC until the 2nd century AD, it was the center of the Kingdom of Dacia. Transylvania was under the control of various tribes residing in the Carpathian basin between the 3rd and 9th centuries AD. Right before it went under Hungarian control, Transylvania was ruled by the Bulgars, a Slavic ethnic group of the Balkans. After Hungarians conquered the Carpathian basin, it became under partial Hungarian control in 1003 AD (Engel, 2005). The settlement of the Hungarians in Transylvania occurred between the late 10th and 13th centuries, but it is still disputed by historians, since Hungarian artifacts were found in Transylvania also from the first half of the 10th century. This settlement processes resulted in the establishment of Transylvanian Hungarian settlements dating back to the era of the Arpad Dynasty. However, the origin of the Székelys (referred to in English and German as Szeklers, in Romanian Secui, in Latin Siculi) is uncertain and still disputed. After World War I, in 1920, Transylvania was annexed to Romania.

The Szeklers are a Hungarian-speaking, Catholic, Hungarian ethnic group. They declared themselves as a special cultural entity with special origin and history. The first written evidence of Szeklers originates from the 12th century, 1116 AD, and traditionally they derive themselves from the Huns (Hermann, 2004). The territory of the Szeklers was first governed under the leadership of the Count of the Székelys (in Latin: Comes Siculorum), who was at the beginning a royal appointee from the Hungarian nobility and was de facto a margrave, which was the military leader of the borderlands. From the 15th century, the military leaders of Transylvania, the voivodes, held the title themselves. The Szeklers were considered a separate ethnic group (natio Siculica) and were a part of the so-called Unio Trium Nationum (“Union of Three Nations”). This was a coalition of three Transylvanian factions. The official censuses with information on the population of Transylvania have been ad hoc conducted since the 18th century. In 1784, Emperor Joseph II called for the first census of the Habsburg Empire, which included Transylvania (Piaschka, 1975). According to that, which stands even nowadays, the population of Transylvania is mixed, including mainly Romanians, Hungarians, Saxons, and Szeklers (Seton-Watson and William, 1933; Stroschein, 2012).

Korond is a Szekler commune comprising five villages with the administrative central village named Korond. It lies in Hargitha County, Transylvania, Romania, deep in the heart of the Székely Land, and its roots go back with the history of the Szeklers to the 12th century. Nowadays, the Szeklers are one of the largest minorities of Europe, from which the majority, 500–700,000 people are living in the Székely Land, Transylvania (Eberhardt, 2003). Korond is traditionally a genetically isolated area, and according to a 2011 census, the commune had a population count of 6135, of which 94% were Hungarian ethnicities (mostly Szeklers), 2.6% were Roma, and 0.3% were Romanians (National Institute of Statistics, 2011). The Szeklers live in the highest population density in the Székely Land, and their name is possibly derived from a Hungarian expression meaning “frontier guards” since they stationed near the Eastern borders of the 1000 years historical Kingdom of Hungary. As mentioned before, historically, the Szeklers derive themselves from the Huns, as after the death of Attila, several of their tribes left Europe; meanwhile, some of them settled in Transylvania (Barta et al., 1994; Hermann, 2004; Egyed, 2013). The non-Szekler Hungarians are from an area located about 60 km from Korond, from the area of Szászrégen and these villages also are traditionally closed communities.

Here we analyzed 24 Szekler samples collected from Korond (in Romanian: Corund), and 24 samples from people identifying themselves as non-Szekler Hungarians living in Transylvanian villages with known history from the age of the Árpád Dynasty (1000–1301 AD). We used the high-density Illumina Infinium Global Screening Array v.3.0 platform to genotype the DNA samples collected from systematically selected individuals and extracted from whole blood samples. We conducted allele frequency correlation-based population stratification, ancestry estimation, and admixture analysis methods as well as haplotype-based ancestry source and homozygosity-by-descent analysis to lay down the foundations of the characterization of Transylvanian Hungarian ethnicities based on genome-wide autosomal marker data. The aim of this study was to assess the extent of isolation of these ethnic groups in order to characterize the population and also to determine the accuracy of our sampling strategy. We also investigated their relationship to contemporary Hungarians of Hungary.

## Materials and Methods

### Datasets

Samples from 24 Szekler individuals living in the Transylvanian village of Korond (Transylvania-living Szeklers—TLS) and another 24 samples obtained from Transylvanian villages (Transylvania-living Hungarians—TLH), having documented history since the age of the Árpád Dynasty (1000–1301 AD), were involved in this study. EDTA-anticoagulated whole blood samples were collected from the participants and served as the source of DNA. The samples were stored below −30°C until DNA isolation and genotyping. Sample collection was based on self-declaration, and was carried out by local community leaders and local pastors. The participants were unrelated, and both genders were represented approximately equally. Their age group was 40–80 years old. Samples were collected exclusively from those persons who could declare with high fidelity that they are Szeklers, or Transylvanian non-Szekler Hungarians. All subjects have documented records back to at least two generations, the parents and grandparents of each participant belong to the same ethnic group and were born in the same area as the respective study participant. The Szekler participants were from Korond, while the non-Szekler Hungarians derived from the Szászrégen (traditional Hungarian settlement name, in Romanian: Reghin) area, Maros (Mureş) county. Within a radius of 15 km, the following villages were the sources of the non-Szekler Hungarian samples: Disznajó (Vălenii de Mureş; 11 subjects), Fickópataka (Fiţcău; *n* = 5), Marosjára (Iara de Mureş, *n* = 6), and Magyaró (Comuna Aluniș, *n* = 2).

A total of 48 samples of Hungarian-speaking ethnic groups from Transylvania were genotyped on the Illumina Infinium Global Screening Array platform (725,831 SNPs). DNA isolation, genotyping, and preliminary quality control were carried out by a third-party service provider (Human Genomics Facility) on the University of Rotterdam in the Netherlands. Additional quality control and data preparation for population genetic analyses were performed domestically applying PLINK1.9 and 2.0 software packages (Purcell et al., 2007; Chang et al., 2015). Illumina identifiers were replaced by reference SNP cluster IDs, and SNPs showing missing genotypes were removed from the data applying the “geno” flag of PLINK1.9. The threshold of missing call frequency was set to 0.1. The SNPs that failed in the Hardy-Weinberg equilibrium tests (*p* = 0.05) were also removed from the data. After the preparation procedures, 665,073 SNPs remained in the dataset.

The participants gave their written informed consent to participate in this study. They all got personal verbal information prior to their signed consent. All samples were anonymized. The study belongs to a series of investigations that—along with the consent form—were approved by the National Ethics Board (ETT TUKEB), and by Regional Ethics Committee of Pécs. The research was conducted according to the principles expressed in the Declaration of Helsinki.

In addition to our data, we also used genome-wide autosomal marker data of populations from the Human Genome Diversity Project whole genomes to increase overlap (genotyped on Illumina 650Y platform) and also considered two datasets (genotyped on Illumina 610K platforms) obtained from the open genotype database of the Estonian Biocentre detailed in Supplementary Table S3 (Cavalli-Sforza, 2005; Rosenberg et al., 2005; Behar et al., 2010; Yunusbayev et al., 2012). We performed on these data the same quality control as described above in the case of TLH and TLS. From the HGDP data, we used the available populations of Europe, the Caucasus area, and South Asia. European groups were the French (*n* = 29), Orcadians (*n* = 16), French Basques (*n* = 24), North Italians (*n* = 13), Sardinians (*n* = 28), Tuscans (*n* = 8), and Russians (*n* = 25). A population from the Caucasus, the Adygei (*n* = 17) was also used. South Asian populations were the Balochi (*n* = 23), Brahui (*n* = 25), Burusho (*n* = 25), Hazara (*n* = 16), Kalash (*n* = 23), Makrani (*n* = 23), Pashtun (*n* = 23), and Sindhi (*n* = 23). We used the Uyghurs (*n* = 10), the Han Chinese (*n* = 44), and the Yoruba samples (*n* = 19) from the HGDP dataset as outgroups. From the data of the Estonian Biocentre, we selected Hungarian samples (*n* = 20) representing the Hungarians living in today’s Hungary (we will refer to them simply as Hungarians from now on) and Romanians (*n* = 16) of contemporary Romania. German samples were also selected from the Estonian data. All the data were tested in preliminary population structure and ancestry analyses such as PCA and ADMIXTURE. German data were filtered according to preliminary PCA, since we discovered that some of the German samples are outliers possessing significant non-West European genetic ancestry. After filtering, 10 German samples remained.

### Population Structure, Ancestry Analysis, and F_st_ Calculations

Population structure analysis was carried out with the SMARTPCA software, which is part of the EIGENSOFT 6.01 software package (Patterson et al., 2006). In these investigations, we used a merged dataset containing all previously listed populations, which contained 578 individuals and 110,734 SNPs. The background linkage disequilibrium in genome-wide marker data can bias both PCA and maximum likelihood estimation methods based on expectation maximization. Therefore, SNPs with strong LD were removed from the data using the “indep-pairwise” pruning command of PLINK 1.9. The r^2^ threshold was set to 0.3, all other parameters were left at their default setting. After the pruning process, 80,056 SNPs were present in the data. We run SMARTPCA with its default settings. Sigma threshold of outlier samples was set to 6.0 and the option “altnormstyle” was set to enabled, which uses the normalization formula presented in Patterson et al. (2006). Besides the calculated eigenvectors and eigenvalues, SMARTPCA also outputs an F_st_ matrix containing the pairwise average allele frequency differentiation values between the analyzed populations. Therefore, Fst calculations were also carried out with the SMARTPCA algorithm.

Ancestry estimation was performed using ADMIXTURE 1.22 software package (Alexander et al., 2009). We calculated probable ancestries with K values from 2 to 10 and cross-validation was also performed in order to find the best K value that fits to our dataset.

### DNA Segment Analyses

DNA segments of interest were identified using Refined IBD 1.02 (Browning and Browning, 2013). Both identical-by-descent (IBD) and homozygous-by-descent (HBD) segment detection were carried out with the same software. These analyses are haplotype-based analyses as opposed to the previous methods, which are allele frequency-based approaches. Therefore, they do not need to be pruned based on background LD. We used the unpruned version of the dataset created for the PCA and ADMIXTURE analysis (*n* = 578; 110,734 SNPs). Genotyping data in binary PLINK format was converted to VCF format using the PLINK/SEQ algorithm (Purcell, 2014). Refined IBD analysis was performed with an IBD scale parameter set to 2.4 according to the recommendations of the developers of the algorithm. Window size was set to 25,000 and window overlap value was 3000. All other parameters were left on its default setting.

Average pairwise IBD sharing was calculated from the output of refined IBD according to the method applied in Atzmon et al. (2010).

$$\text{Average}\;\text{share}=\frac{\sum_{\text{i}=1}^{\text{n}}\sum_{\text{j}=1}^{\text{m}}{\text{IBD}}_{\text{ij}}}{\text{n}\times \text{m}}$$

In the equation, IBD_ij_ is the length of the IBD segment shared between the individuals i and j from populations I and J, and n, m are the number of individuals in populations I and J.

HBD segment counts were also calculated from the output data of the same refined IBD run. Length and number of shared IBD segments and HBD segments were investigated by calculating averages and statistics in the form of boxplots.

### The 4-Population Tests

Four-Population tests were applied to check if the relationship of investigated Hungarian-speaking groups, the Szeklers from Korond and Transylvanian Hungarians with Hungarians are well detectable. The purpose of this method was to test the assumed connection of TLH and TLS to Hungarians. It was carried out with the 4-population test algorithm of ADMIXTOOLS 4.1, which is implemented in this software package as D-statistics (Green et al., 2010; Patterson et al., 2012). In this investigation, the same unpruned dataset was used which we applied previously in the IBD analysis. We used the following four populations for the tests: Yoruba, the two groups in the focus of this study, Hungarians, and Romanians. We also checked the setups with Han Chinese and Adygei as outgroups instead of the Yorubas. The 4-population test uses a hypothetical unrooted phylogenetic tree [(W) (X)] [(Y) (Z)], where W, X and Y, Z groups are supposed to be related. If the Z-score of the 4-population test is not significant, then the results are not violating this assumption. If the Z-score is significantly positive (greater than or equal to 3), then W, Y or X, Z. If the Z-score happens to be significantly negative (less than or equal to −3), then W, Z or X, Y are related. Here the setup of the unrooted phylogenetic trees were: [(Yoruba) (Study focus groups) (Hungarians) (Romanians)] and [(Han Chinese) (Study focus groups) (Hungarians) (Romanians)] and [(Adygei) (Study focus groups) (Hungarians) (Romanians)].

## Results

### Relationship of the Transylvanian Samples to European and Worldwide Populations

**FIGURE 1:**
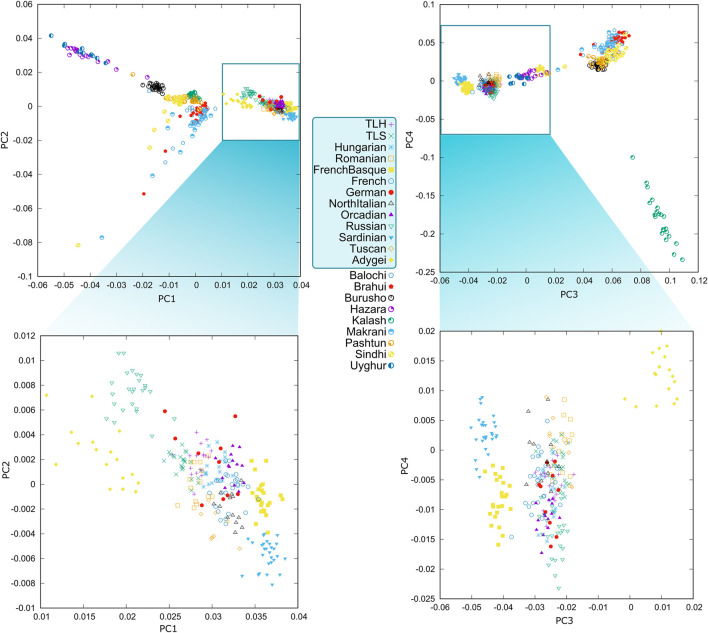
PCA results plotted on the first four eigenvectors. Eigenvalues of the first four principal components were 20.711, 14.184, 6.219, and 3.243, respectively. PCA featuring only 13 populations is the result of the same analysis plotting only the respective populations. TLH—Transylvania-living Hungarians; TLS—Transylvania-living Szeklers.

Both PCA and ADMIXTURE analysis showed that the investigated Szeklers and Transylvanian Hungarians were relatively homogeneous and clustered tightly together and close to Hungarians and Romanians (Figure 1). These populations show a quite high similarity to each other on PCA and on the ADMIXTURE graph, referring to their very strong common history.

The investigated populations are clustered between South and East Europe with Hungarians and Romanians mirroring their actual geographical positions. Europe and the Caucasus are well separated from South Asian populations. Some of the South Asian ethnicities split into two groups depending on whether they have significant East Asian or African derived ancestry components. In this investigation, Han Chinese represented the Eastern component and Yoruba represented the African component, which are clearly separated from each other and from European and South Asian populations. On the contrary, Europeans do not show such a tendency. Han Chinese and Yoruba groups were not incorporated on the PCA graphs due to the large plotting distances, however, the complete PCA can be found in the Supplementary Figure S1.

**FIGURE 2:**
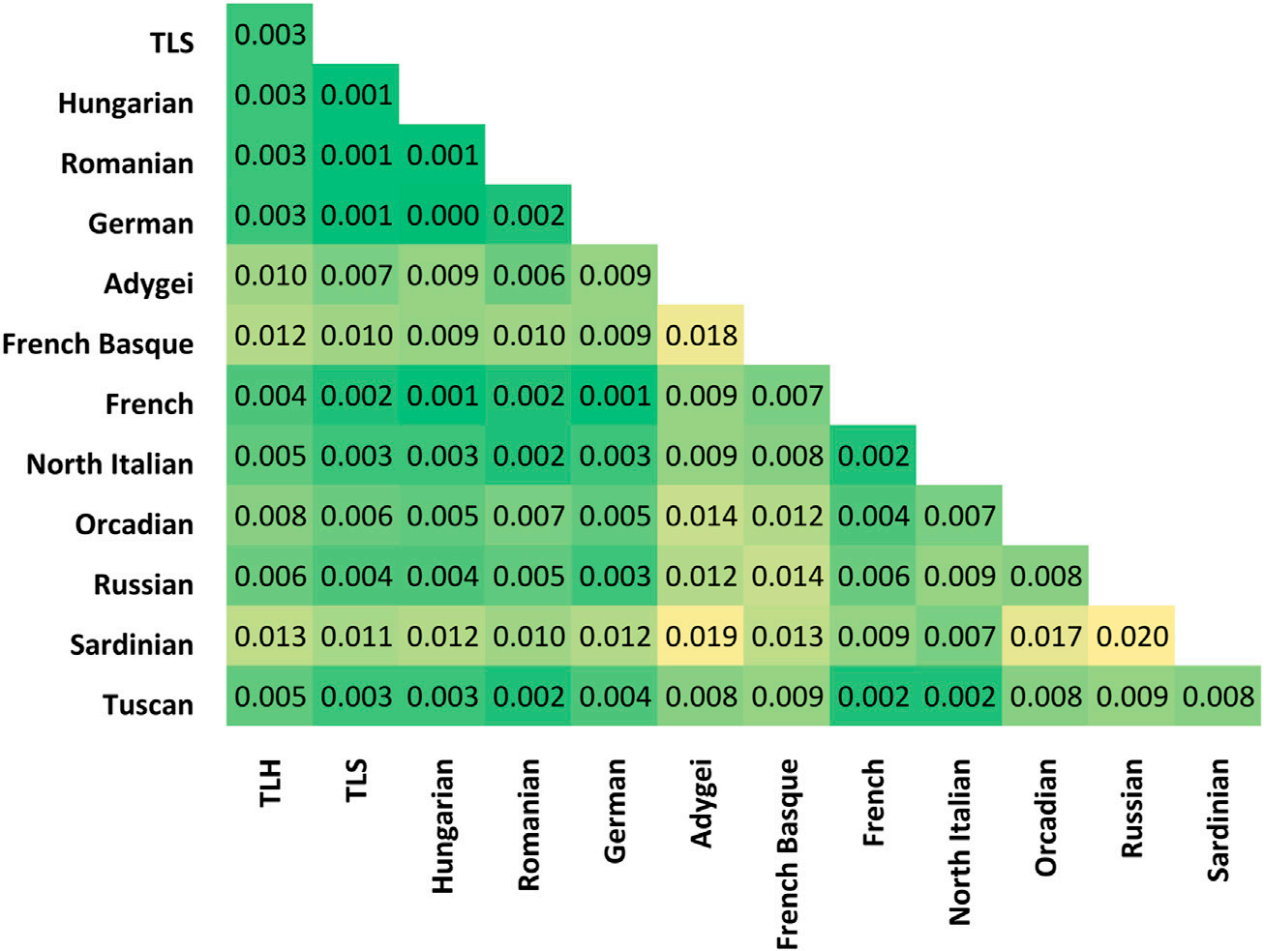
F_st_ calculation results.

F_st_ calculations also show that investigated populations are close to each other, with neighboring populations, and with Germans. This observed similarity with Germans might be formed through their admixture with the Transylvanian Saxon population and in the case of Hungarians with direct admixture with Swabian immigrants (Figure 2). It can also be observed on the PCA figure, since TLH, TLS, and Hungarian clusters are significantly overlapping with Germans. TLH, TLS, Hungarians, and Romanians have the highest F_st_ with geographically isolated groups like Orcadians and Sardinians and genetically isolated populations like French Basques. F_st_ values also reflect the geographical distances, since Adygei samples have a relatively high F_st_ with the four groups, the TLH, TLS, Hungarians, and Romanians. It can also be observed that TLH samples have the highest F_st_ with all European groups compared to TLS, Hungarians, and Romanians. F_st_ values of TLS is most similar to those of Romanians. Hungarians—along with TLH—tend to have lower F_st_ with West Europeans, Romanians have lower F_st_ with East and South Europeans. According to the average pairwise allele frequency differentiation values, it looks like that TLH is most similar to Hungarians in terms of F_st_ differences considering the various European populations. They share a same pattern and are just farther away from them genetically than Hungarians.

**FIGURE 3:**
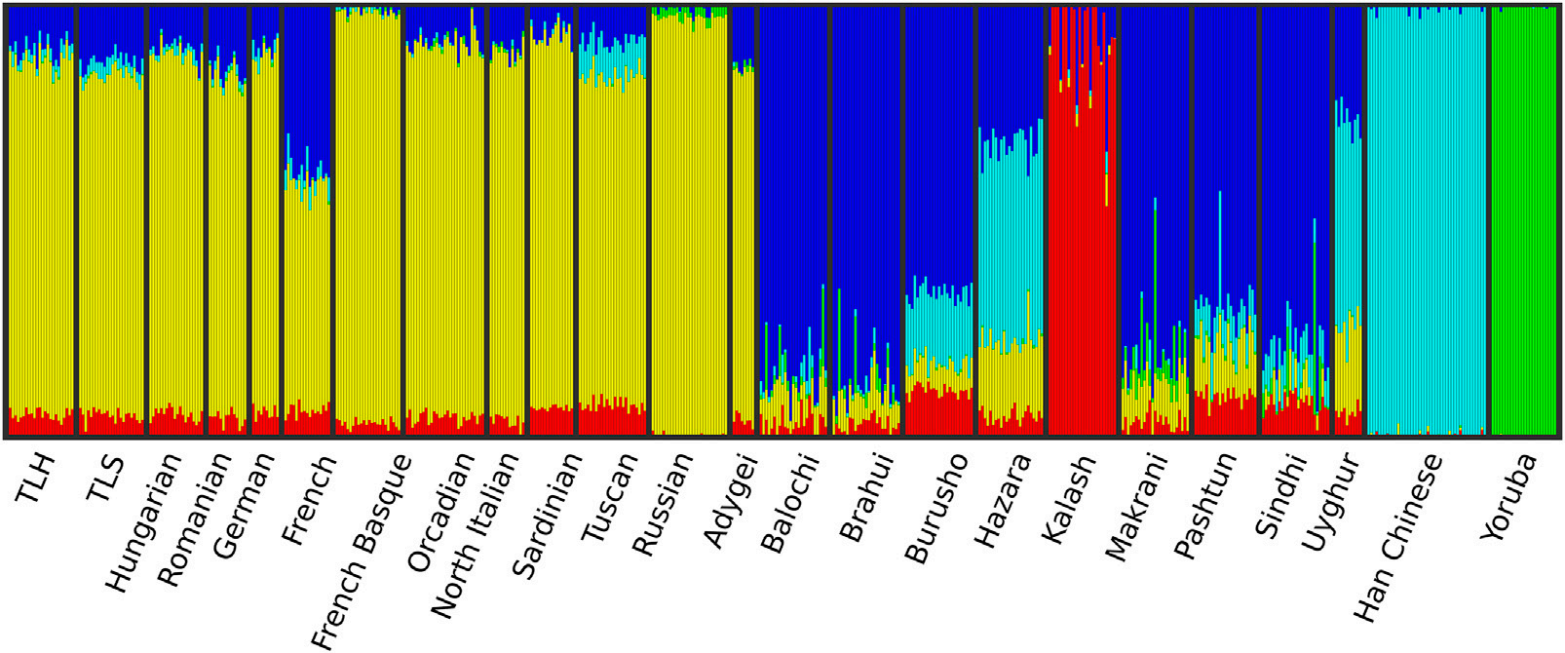
Admixture analysis results of the investigated populations with K = 5 hypothetical ancestral groups.

The cross-validation error of the ADMIXTURE analysis was the lowest at K = 5 hypothetical ancestral groups (Supplementary Figure S2). The analysis supported the results of the PCA and showed also that French Basques and, in the case of the PCA, the Sardinians are separated from other European populations. This can be explained due to the well-known strong genetic isolation of the Basque and Sardinian populations (Figure 3).

### Seeking for the Source of Ancestry

**FIGURE 4:**
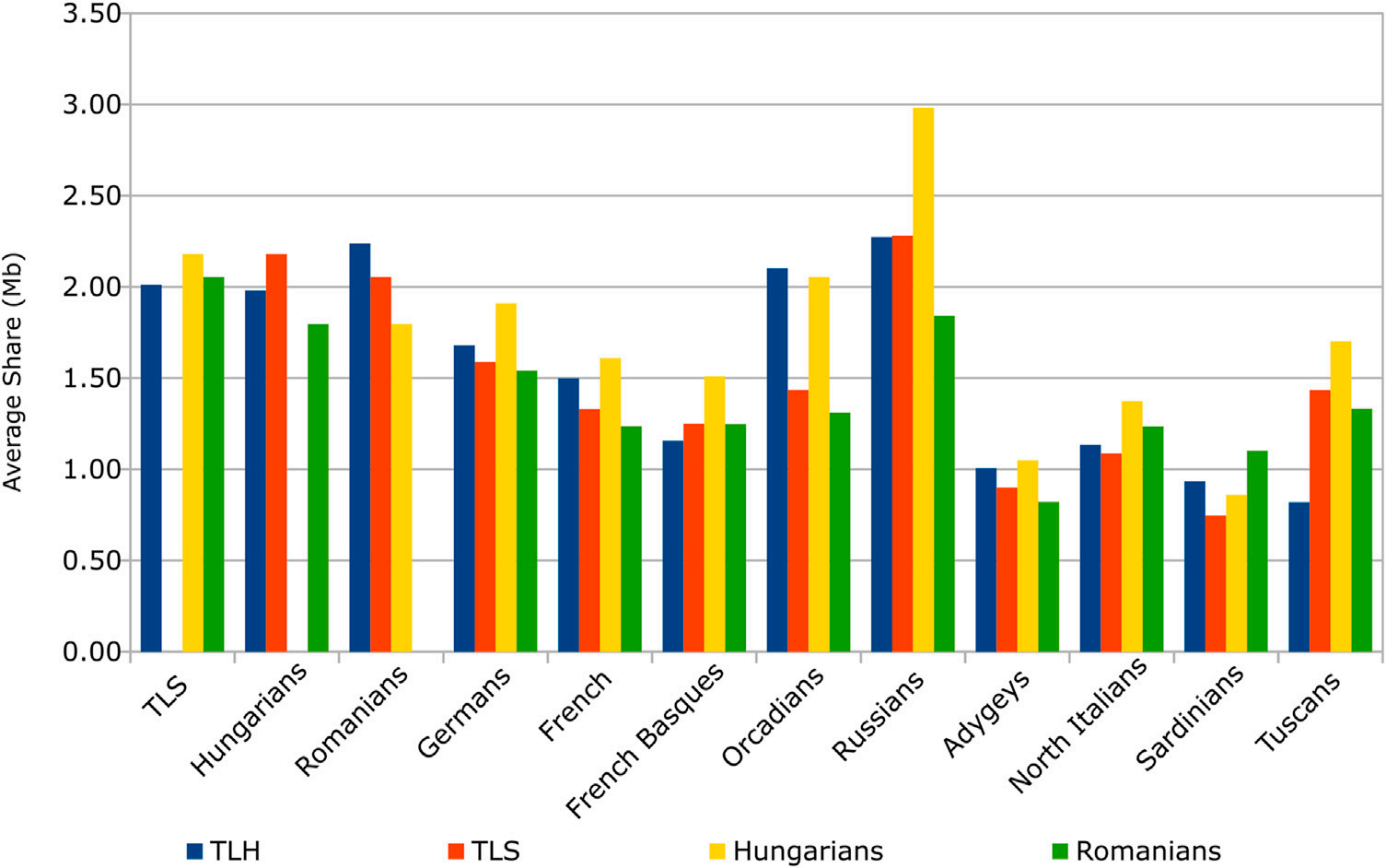
Average pairwise IBD sharing between investigated populations.

According to the average pairwise IBD sharing results, the relationship of TLS and TLH to Hungarians of contemporary Hungary is varied. TLS and TLH both show similarities to Hungarians in certain aspects. Our investigation showed that non-Szekler Transylvanian Hungarians have more similarity to Hungarians in the case of ancestry derived from West Europe. TLS, on the other hand, shares more similarities in South European-derived ancestry with Hungarians. Hungarians have a much more significant East European ancestry component than either TLS or TLH (Figure 4; Supplementary Table S1). These can also be observed on the boxplot diagrams of Supplementary Figure S3, visualizing the IBD segment length distribution of European populations.

Average sharing results show that Hungarians are somewhat closer to TLS than TLH. We know that Hungarians have been admixed heavily with immigrating populations from the age of the Árpád Dynasty until today. This might also suggest that TLH is a somewhat more isolated population and is less affected by admixture with other Transylvanian ethnic groups. Examinations regarding the average length of IBD segments shared between pairs of individuals do not show any recognizable pattern. Analyzing the average number of IBD segments between pairs of individuals pointed out the similarity of Hungarians, TLS, and TLH. However, it also shows that populations in the Transylvanian region, including Romanians, are in general less admixed with West Europeans and also with Russians (Supplementary Figures S4, S5).

### Autozygosity Analysis

**FIGURE 5:**
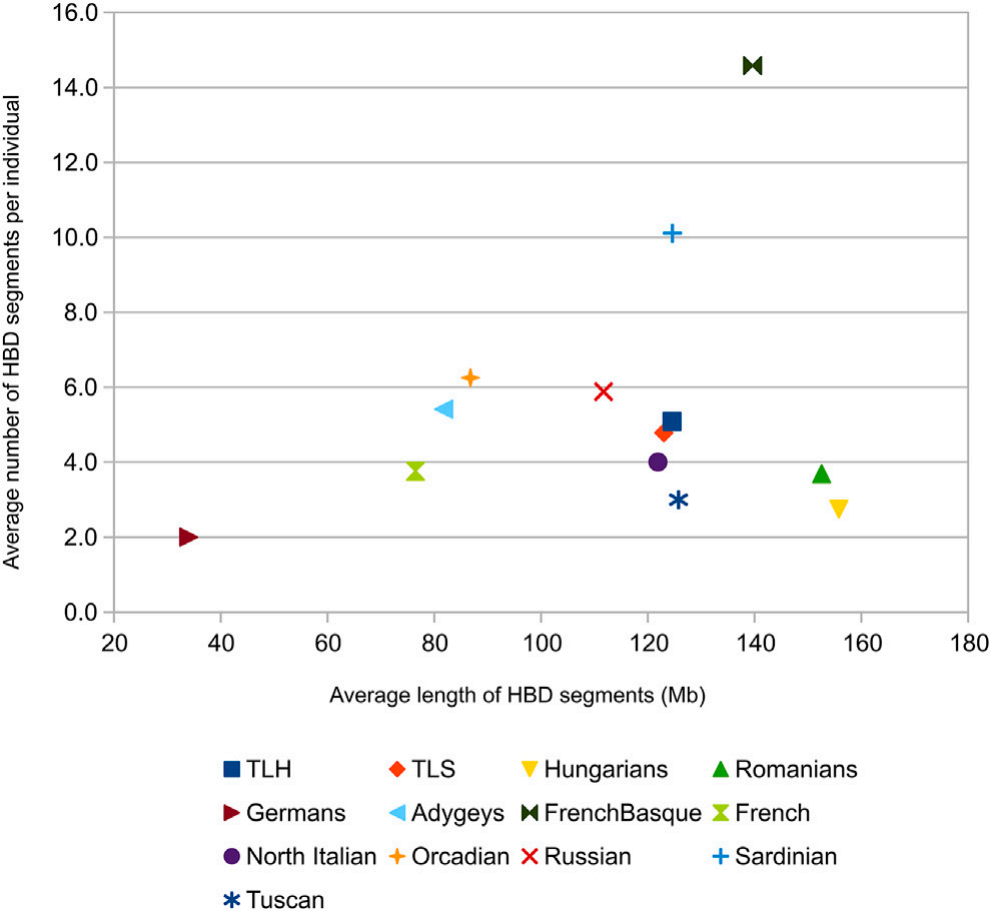
Homozygous-by-descent segment analysis results.

According to the HBD segment analysis, as expected, French Basques, Sardinians, and Orcadians showed the highest extent of autozygosity indicating their strongly isolated nature. Szeklers and Transylvanian Hungarians show a higher autozygosity relative to most European populations, therefore, also higher than the autozigosity observed in Hungarians and Romanians (Figure 5; Supplementary Table S2), which could indicate their relatively higher genetic isolation. Investigating the variation of HBD segment number per individual from all groups, we can observe that TLH and TLS have indeed higher numbers of HBD segments compared to Hungarians, Romanians, and most West European populations. HBD segment length examination per individual shows that HBD length is highly variable in the case of most groups, except of Hungarians, Romanians, Germans, and Tuscans (Supplementary Figure S6).

### Relationship to Hungarians Living in Contemporary Hungary

The 4-population tests revealed that TLS and TLH are strongly related to contemporary Hungarians living in Hungary despite the many admixture events Hungarians went through during the centuries. The results were similar in all three cases applying the three distinct ethnic groups as outgroup populations. Z-scores showed significant negative values indicating that W,Z or X,Y groups are related to each other on the hypothetical unrooted phylogenetic tree (Table 1). In our case, the close relationship of X and Y, TLS and TLH with Hungarians is more likely. All calculations showed significantly high negative Z-scores, however, it was most significant in the case we used the Adygei from the Caucasus region as an outgroup.

**TABLE 1:** 4-population test results.

Outgroup	Pop1	Pop2	Pop3	D-stats	Z-score
W	X	Y	Z		
Yoruba	TLH	Hungarian	Romanian	−0.0075	−9.243
Yoruba	TLS	Hungarian	Romanian	−0.0074	−9.418
HanChinese	TLH	Hungarian	Romanian	−0.0071	−8.789
HanChinese	TLS	Hungarian	Romanian	−0.007	−8.668
Adygei	TLH	Hungarian	Romanian	−0.0041	−9.715
Adygei	TLS	Hungarian	Romanian	−0.004	−9.895

## Discussion

Population structure analysis by PCA and ancestry estimation with ADMIXTURE suggested that TLH and TLS groups are homogeneous, and they showed similar genetic ancestry. Placing these populations in a Eurasian perspective, they were shown to be a well-defined, tightly clustered group, suggesting no signs of recent admixture with populations from other regions considered in these tests.

The DNA segment analyses using identity-by-descent segment discovering tools pointed out that average pairwise IBD sharing results are similar in TLS, TLH, Hungarians, and Romanians. However, TLS samples had a higher average sharing with the Hungarians. Average sharing results also showed that the investigated Szeklers from Korond might be somewhat less isolated than the inhabitants of Árpád Age villages. This was confirmed by our autozygosity analysis as well. Our IBD calculations pointed out that Romanians differ from Hungarians, TLH, and TLS in the number of detected IBD segments between pairs of individuals, suggesting that Hungarians, TLH, and TLS have a common history. Although this tendency is much more significant in the case of TLH, they also show similar tendencies than of Hungarians regarding the relationship with West Europeans. This has been also indicated earlier by the fixation index values. Our investigations of haplotype-based DNA segment analysis concluded that these two areas, the commune of Korond and the people of the surrounding villages of Szászrégen, are noticeably isolated compared to most European populations, which could have helped to retain the strong relationship with the people of contemporary Hungary even though the contemporary Hungarian population is admixed, showing strong sources of ancestry from both West and East Europe. The source of West European ancestry might be primarily the Germans through the Swabian immigration mentioned before.

The 4-population tests also showed that these Hungarian-speaking ethnic groups, the Szeklers from Korond and Transylvanian Hungarians, are strongly related to Hungarians living in Hungary.

Earlier genetic studies involving Hungarians and Hungarian-speaking ethnicities outside of contemporary Hungary are based on Y chromosomal and mitochondrial DNA haplogroup analyses and gene frequency analysis of blood groups and red cell enzymes coding nuclear loci (Guglielmino et al., 2000; Brandstatter et al., 2007; Biro et al., 2015). The results of these investigations showed that Hungarians and Hungarian-speaking ethnicities of Romania have significant Middle Eastern ancestry from the Iranians and Turks besides the Slav and German derived ancestry components. Hungarian-speaking ethnicities of Transylvania also have a higher rate of Turkic ancestry than Hungarians living in Hungary. Certain studies pointed out that Hungarian ethnicities in Transylvania also have Central Asian genetic elements, with the highest extent in the case of the Szeklers, which was 7.3% (Biro et al., 2015). That could also imply that Hungarians from ancient Transylvanian villages and the Szeklers are a rather isolated population with less admixture between the neighboring ethnicities involved. The isolated nature of Szeklers and Transylvanian Hungarians could preserve these Central Asian and Turkic roots. This Central Asian genetic heritage needs further investigations in order to shed light on its origin, which could point out indirect transmission through Slavic groups, but also could strengthen the Central Asian origin of Szeklers on a genetic basis. One of our previous studies investigated Csango samples from Bacau county, Romania (Ádám et al., 2020). Csangos are a Transylvanian Hungarian ethnic group similar to Szeklers with an even more isolated nature and showed to have a unique Turkic ancestry from Central European and Siberian Turkic populations. These results, regarding the significant Turkic ancestry estimated in Csangos, are in line with the previously described Central Asian Turkic ancestry and is even more significant than the Turkic ancestry proportion reported previously in the Szeklers. Investigating the Szeklers in a similar manner with subsequent studies could help to discover their nature. We could get closer to learning about their exact origins and could shed light on their connection with other Hungarian ethnic groups of Transylvania such as the Csangos.

In summary, we can conclude that Szeklers from the area of Korond and non-Szekler Transylvanian Hungarians are homogeneous, and more isolated than the continental population of Europe, especially in the case of the Western Europeans. Our analyses show that their genetic connection to Hungarians is still well detectable not only using methods based on allele frequency calculations, but also results of haplotype-based methods suggest this conclusion.

Our study is the first genome-wide autosomal marker-based analysis of Szekler population samples, and of samples from Transylvania living non-Szekler Hungarian population living in settlements established in the age of the Árpád Dynasty. With our investigation, we described the genetic makeup of Szeklers, assessed their degree of isolation, and found that they can be separated from Transylvanian Hungarians using genome-wide data. We also described their connection with Hungarians, Romanians, and European populations. Subsequent research should aim at the study of their assumed Central Asian and East Asian Turkic ancestry.

## Data Availability

The original contributions presented in the study are included in the article/Supplementary Material, further inquiries regarding the Szekler data can be directed to the corresponding authors.
