# Supplementary material for: Genome-Wide Marker Data-Based Comparative Population Analysis of Szeklers From Korond, Transylvania, and From Transylvania Living Non-Szekler Hungarians
Source: Front Genet. 2022 Mar 28;13:841769. doi: 10.3389/fgene.2022.841769 (PMC9000985; doi:10.3389/fgene.2022.841769)
Supplement: Supplementary file 1 [file DataSheet7.PDF]

Supplementary Table 2. HBD segment calculation results.

	average number of hbd segments	average length of hbd segments (Mb)
**TLH**	5.1	124.54
**TLS**	4.8	123.00
**Hungarians**	2.8	155.79
**Romanians**	3.7	152.57
**Germans**	2.0	34.00
**Adygeys**	5.4	81.76
**FrenchBasque**	14.6	139.62
**French**	3.8	76.48
**North Italian**	4.0	121.90
**Orcadian**	6.3	86.77
**Russian**	5.9	111.72
**Sardinian**	10.1	124.62
**Tuscan**	3.0	125.76
