# Supplementary figures and images for: Genome-Wide Marker Data-Based Comparative Population Analysis of Szeklers From Korond, Transylvania, and From Transylvania Living Non-Szekler Hungarians
Source: Front Genet. 2022 Mar 28;13:841769. doi: 10.3389/fgene.2022.841769 (PMC9000985; doi:10.3389/fgene.2022.841769)

**Supplementary Figure 2.** Cross-validation results of ADMIXTURE analysis with K=2 to K=10.

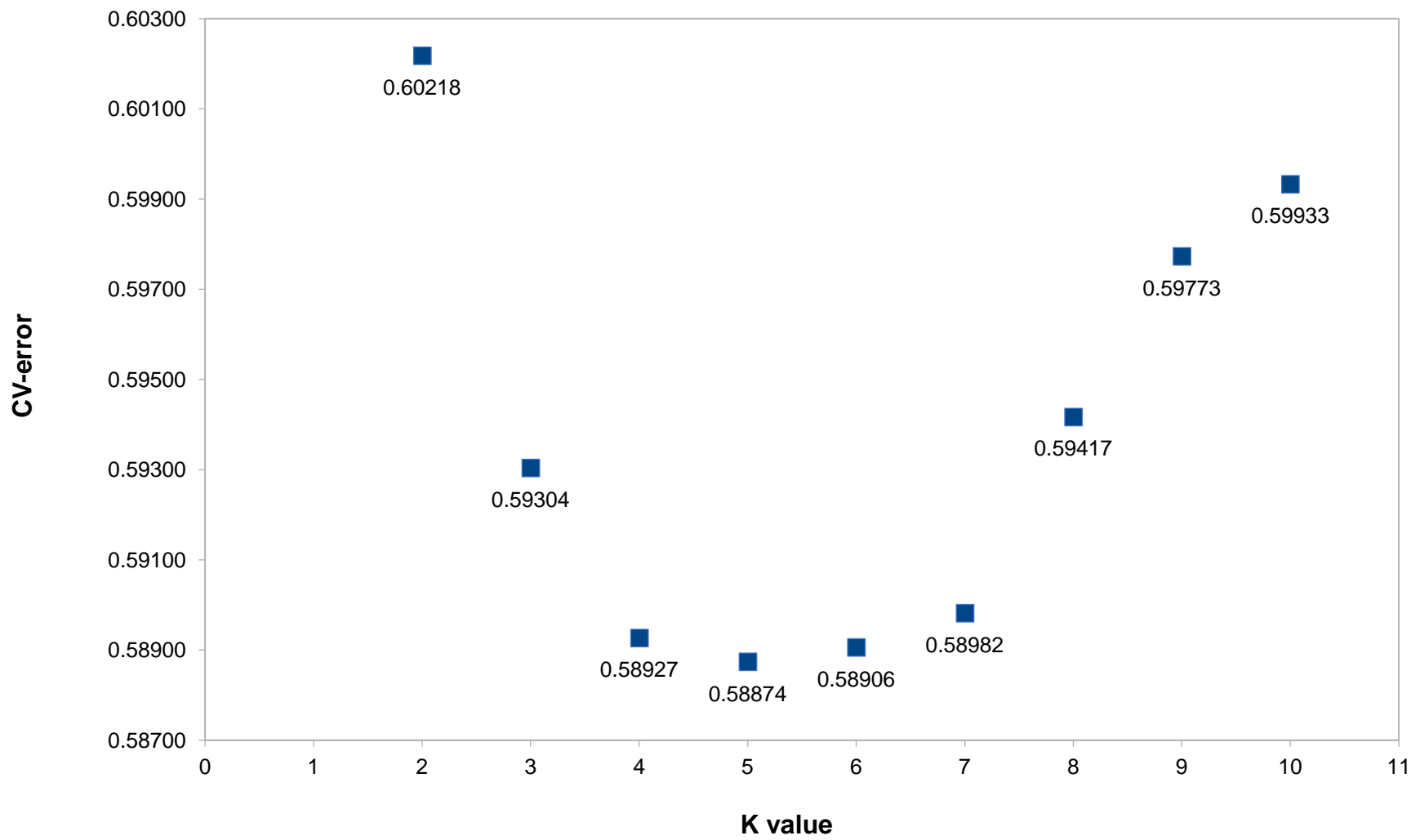

Supplement: Supplementary file 2 [file DataSheet2.PDF]
