# Supplementary material for: Genome-Wide Marker Data-Based Comparative Population Analysis of Szeklers From Korond, Transylvania, and From Transylvania Living Non-Szekler Hungarians
Source: Front Genet. 2022 Mar 28;13:841769. doi: 10.3389/fgene.2022.841769 (PMC9000985; doi:10.3389/fgene.2022.841769)
Supplement: Supplementary file 5 [file DataSheet9.PDF]

Supplementary Table 3. Availability of datasets analyzed in this study.

Dataset	Source	Availability
**Szeklers and non-Szekler Transylvanian Hungarians**	Department of Medical Genetics, Medical School, University of Pécs	Available upon reasonable request from the corresponding authors. Data request can be initiated by contacting the corresponding author, melegh.bela@pte.hu
**Human Genome Diversity Project - HGDP-CEPH Human Genome Diversity Cell Line Panel**	Stanford University (HudsonAlpha Genome Sequencing Center)	URL: https://www.hagsc.org/hgdp/
**Estonian Biocentre - Yunusbayev et al. 2012**	Public online repository of the Estonian Biocentre	URL: https://evolbio.ut.ee/caucasus/
**Estonian Biocentre - Behar et al. 2010**	Public online repository of the Estonian Biocentre	URL: https://evolbio.ut.ee/jew/
