# Supplementary material for: Genome-Wide Marker Data-Based Comparative Population Analysis of Szeklers From Korond, Transylvania, and From Transylvania Living Non-Szekler Hungarians
Source: Front Genet. 2022 Mar 28;13:841769. doi: 10.3389/fgene.2022.841769 (PMC9000985; doi:10.3389/fgene.2022.841769)
Supplement: Supplementary file 6 [file DataSheet3.PDF]

Supplementary Table 1. Average pairwise IBD share calculations

	TLH	TLS	Hungarians	Romanians
**TLS**	2.01	-	2.18	2.05
**Hungarians**	1.98	2.18	-	1.80
**Romanians**	2.24	2.05	1.80	-
**Germans**	1.68	1.59	1.91	1.54
**French**	1.50	1.33	1.61	1.24
**French**				
**Basques**	1.16	1.25	1.51	1.25
**Orcadians**	2.10	1.44	2.05	1.31
**Russians**	2.27	2.28	2.98	1.84
**Adygeys**	1.01	0.90	1.05	0.82
**North Italians**	1.13	1.09	1.37	1.23
**Sardinians**	0.93	0.75	0.86	1.10
**Tuscans**	0.82	1.44	1.70	1.33
