# Supplementary material for: Genome-Wide Marker Data-Based Comparative Population Analysis of Szeklers From Korond, Transylvania, and From Transylvania Living Non-Szekler Hungarians
Source: Front Genet. 2022 Mar 28;13:841769. doi: 10.3389/fgene.2022.841769 (PMC9000985; doi:10.3389/fgene.2022.841769)

Supplementary Figure 1. PCA results featuring all populations implemented in the analysis. Eigenvalues of the first four principal components were 20.711, 14.184, 6.219 and 3.243, respectively.

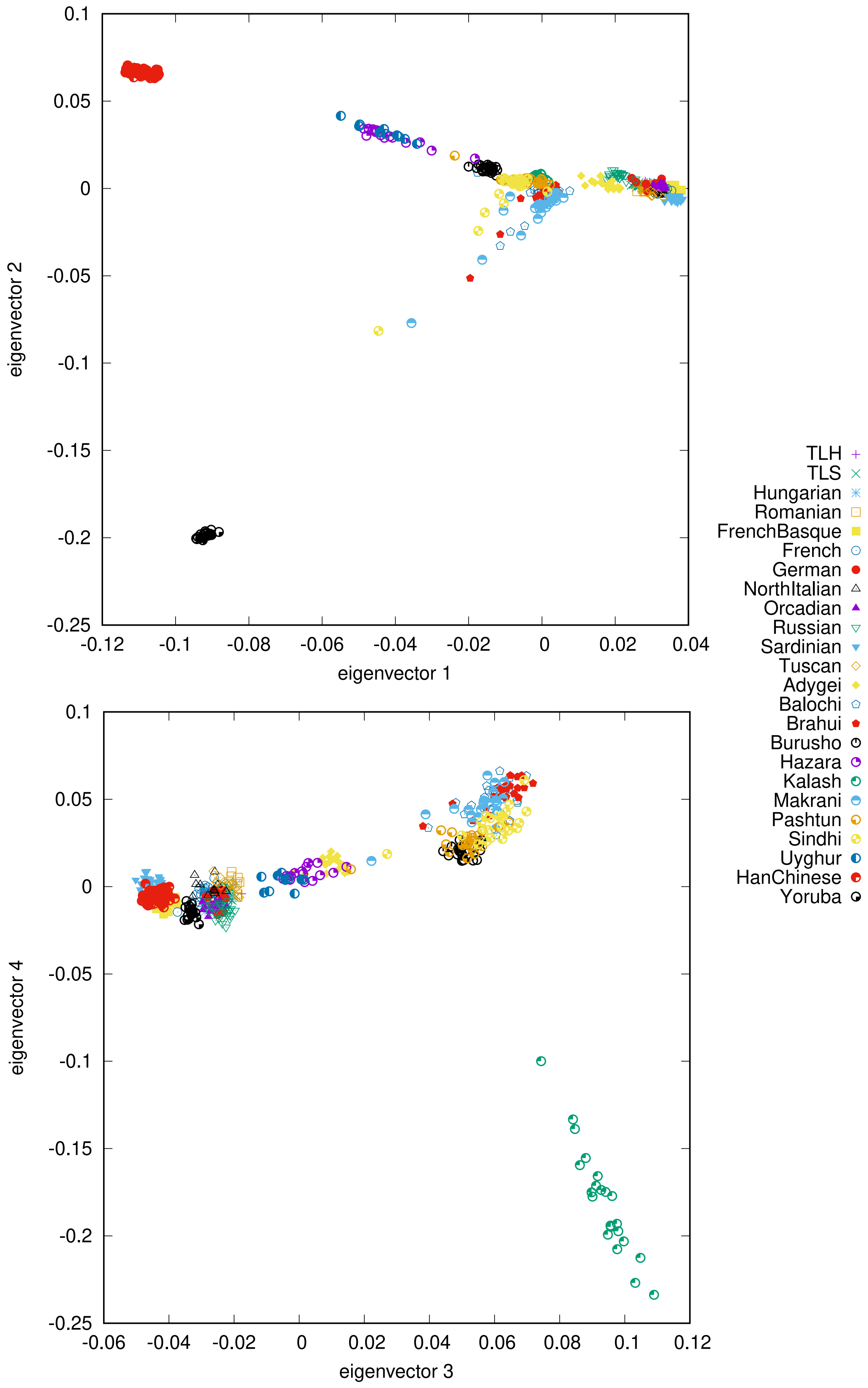

Supplement: Supplementary file 7 [file DataSheet1.PDF]
