# Supplementary material for: Genome-Wide Marker Data-Based Comparative Population Analysis of Szeklers From Korond, Transylvania, and From Transylvania Living Non-Szekler Hungarians
Source: Front Genet. 2022 Mar 28;13:841769. doi: 10.3389/fgene.2022.841769 (PMC9000985; doi:10.3389/fgene.2022.841769)

Supplementary Figure 4. Average length of IBD segments shared between pairs of individuals from investigated groups.

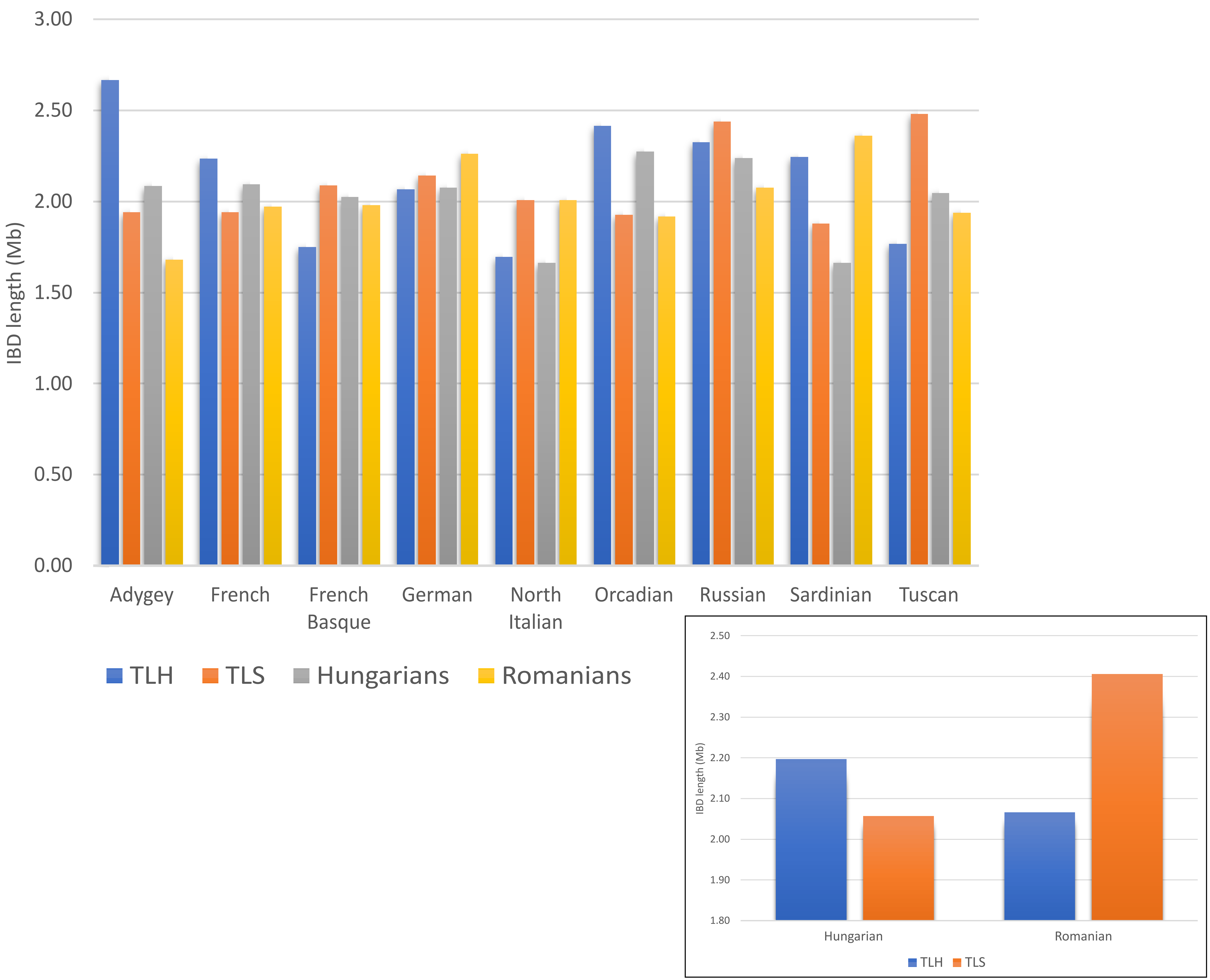

Supplement: Supplementary file 8 [file DataSheet5.PDF]
