# Supplementary material for: Genome-Wide Marker Data-Based Comparative Population Analysis of Szeklers From Korond, Transylvania, and From Transylvania Living Non-Szekler Hungarians
Source: Front Genet. 2022 Mar 28;13:841769. doi: 10.3389/fgene.2022.841769 (PMC9000985; doi:10.3389/fgene.2022.841769)

Supplementary Figure 6. Variation of HBD segment length and number per individual from the investigated populations. (a) Variation of HBD segment length (b) Variation of HBD segment number.

(a)

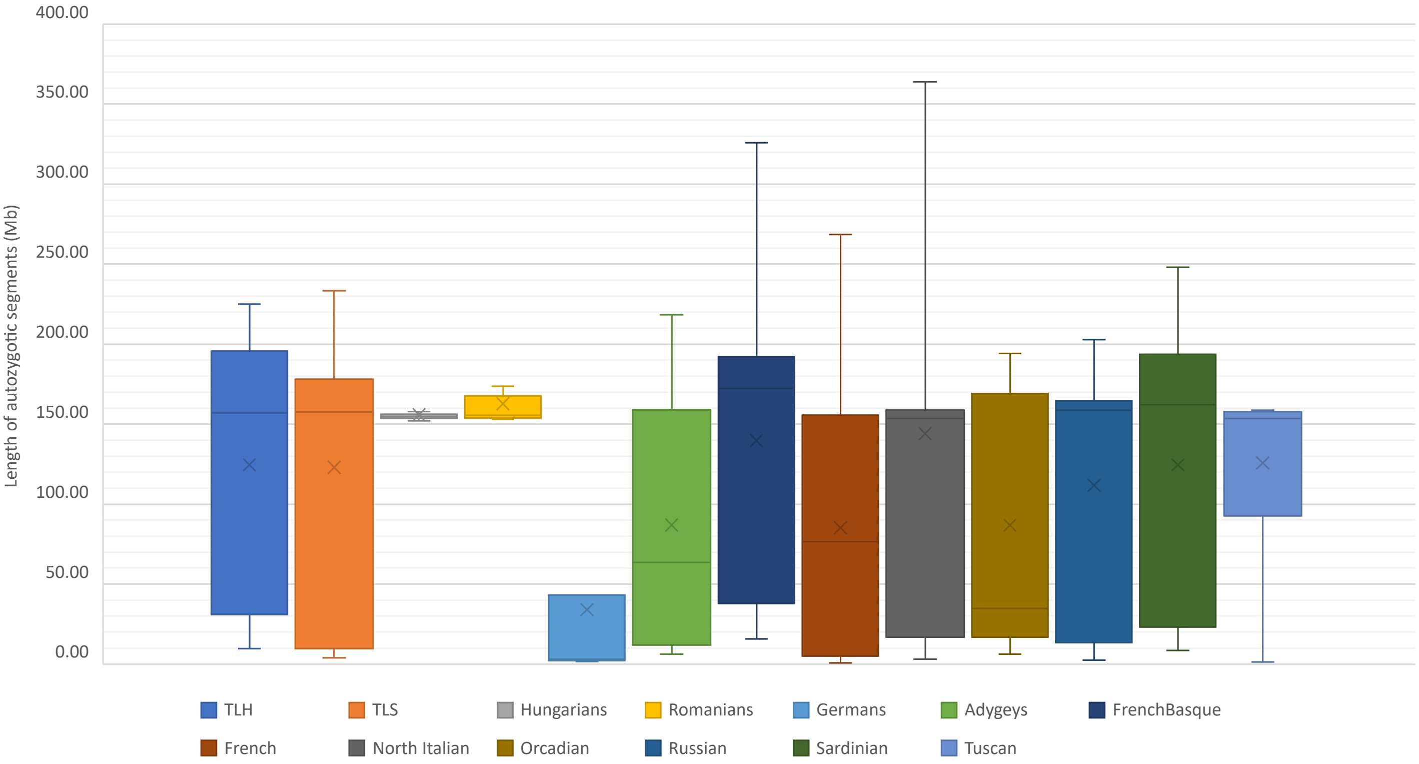

(b)

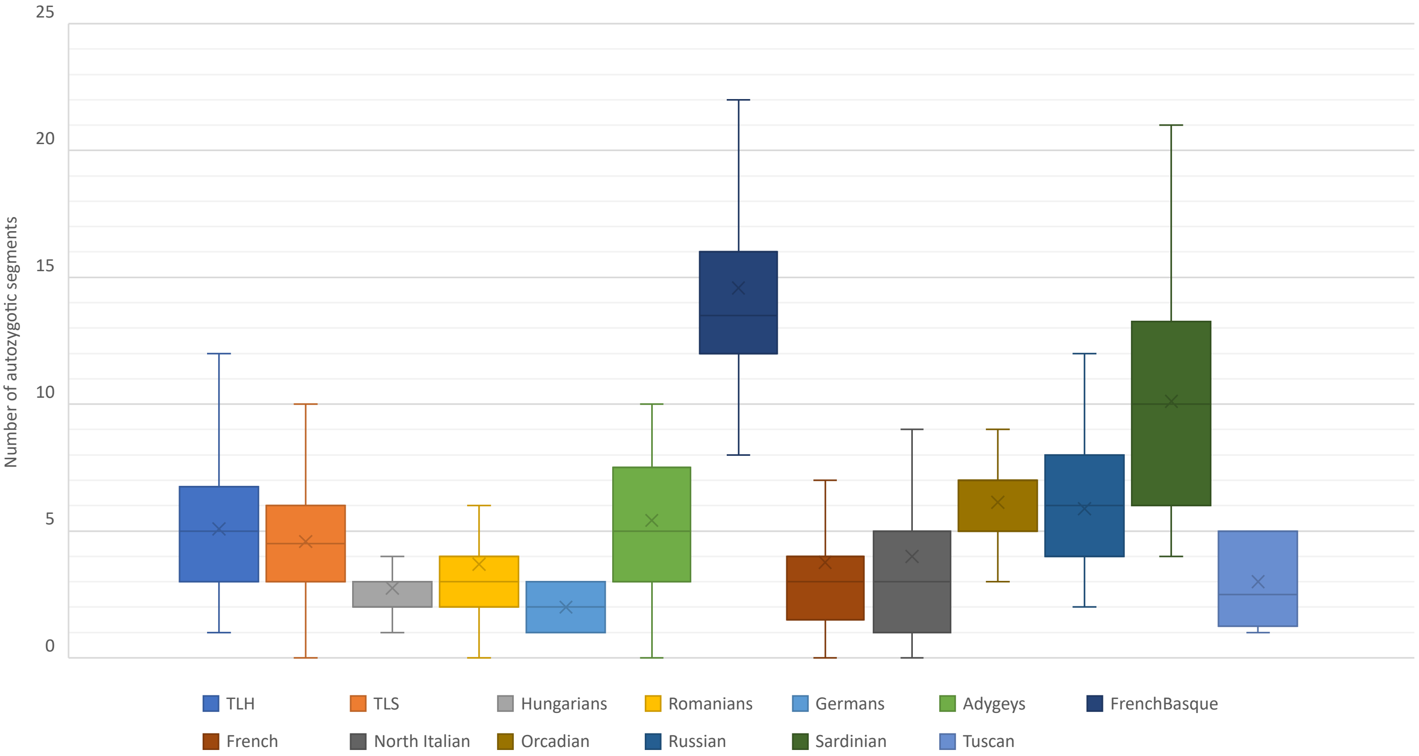

Supplement: Supplementary file 9 [file DataSheet8.PDF]
